# AGEISM EVERYWHERE ALL AT ONCE: DIFFERENT MANIFESTATIONS AND RESEARCH APPROACHES

**DOI:** 10.1093/geroni/igae098.0011

**Published:** 2024-12-31

**Authors:** Alison Chasteen

**Affiliations:** Chair

## Abstract

In the over 50 years since ageism was defined, the impact of age prejudice has never been clearer, with the increased expression of age bias both during and after the COVID-19 pandemic. This symposium aims to highlight the pervasive nature of ageism through a range of methodological approaches, including in-depth interviews, social media scraping, and experimental surveys, to underscore the ubiquity of ageism in contemporary society. We delve into various facets of ageism, exploring its manifestations in ageist group labels, media representations, beauty standards, and even its correlation with suicidal ideation. We also investigate ways to challenge ageist comments through confrontation. Vale assesses the impact of using ageist terms such as ‘elderly’ and ‘senior citizen’ on perceptions of older people. Fraser and colleagues explore online reactions to “The Golden Bachelor,” the first older adult-focused version of the reality tv show. Soulie et al. investigate older women’s responses to ageist beauty norms, including when they accept or resist them. Gendron and colleagues examine the role of internalized ageism in suicidal ideation among older people. Gans et al. test whether reactions to ageism confrontation vary by the intersecting identities (gender and race) of the older adult confronter as well as by the confrontation technique that is used. Altogether, these talks shed light on the multifaceted nature of ageism in North American society and offer valuable insights into strategies for combatting age bias. International Association of Gerontology & Geriatrics North American Regional Committee Sponsored Symposium

